# Analgesia for labour pain – analysis of the trends and associations in the Grampian region of Scotland between 1986 and 2001

**DOI:** 10.1186/1471-2393-6-14

**Published:** 2006-04-19

**Authors:** Sohinee Bhattacharya, Tao Wang, Fiona Knox

**Affiliations:** 1 Dugald Baird Centre for Research on Women's Health, University of Aberdeen, Aberdeen, UK; 2 Department of Public Health, University of Aberdeen, Aberdeen, UK; 3 Department of Anaesthesia, Grampian University Hospitals Trust, Aberdeen UK

## Abstract

**Background:**

Although intrapartum analgesia has been in use since Victorian times, there have been few attempts to study its usage from routinely collected data. This population based epidemiological study aimed to analyse retrospective data on the distribution of different types of labour analgesia used by women in the Grampian region of Scotland between 1986 and 2001 in order to examine time trends and associations.

**Methods:**

Data records on all deliveries occurring in the years 1986 to 2001 were extracted from the Aberdeen Maternity and Neonatal Databank.

The rates of the use of epidural, opioid and Entonox or no analgesia for pain relief in labour in each year were calculated.

Maternal, pregnancy, labour and delivery characteristics were compared among the users of three different analgesics by univariate and multivariate analyses.

**Results:**

A total of 81,418 deliveries were analysed. Of these, 12,659 (15.5%) women had epidural, 33,819 (41.5%) had used opioids and 26,974(33.1%) received either Entonox or no analgesia at all. The women who received epidural analgesia were younger, shorter and heavier and had larger babies (OR = 1.05, 95% CI 1.01, 1.08). Three quarters of them were primigravidae and had longer periods of gestation. They were also more likely to have suffered pregnancy related complications (OR = 2.11, 95% CI 1.8, 2.4). Labour was more likely to have been induced (OR = 2.8, 95% CI 2.6, 2.9) and to have lasted longer in this group of women. Women in this group were 5 times more likely to have an instrumental delivery (95% CI 4.9, 5.1) and 7 times more likely to have a Caesarean section (95% CI 5.7, 9.3).

**Conclusion:**

Non epidural analgesia was found to be the most popular choice for pain relief in labour in the Grampian region between 1986 and 2001, although an increase in the uptake of epidural services is starting to occur. The type of labour analgesia used is associated with the epidemiological characteristics of the women's pregnancy, labour and delivery.

## Background

A lot of anaesthetic has flowed in the labour wards since James Simpson in 1847 used ether to provide analgesia for a vaginal delivery and John Snow administered chloroform to Queen Victoria for the birth of her eighth and ninth children. Yet more than a century later, the debate over the choice and safety of the different types of analgesia for labour pain continues. While epidurals provide better pain relief their effect on the progress of labour and instrumental and caesarean delivery remains controversial [1,2].

Despite the safety and acceptability of regional analgesia, an NHS wide survey carried out for all deliveries in 2002–2003 showed that only a third of all deliveries took place under regional anaesthesia/analgesia [3]. This is in sharp contrast to the USA, where 60% of labouring women choose epidural analgesia. The CRAG report [4] suggested an East-West divide in the use of epidurals in Scotland, ranging from 15% to 30%. This was partly explained by the variation in availability of epidural analgesia in the different obstetric units. Where there was full availability, the variation was accounted for by the differential uptake of the services by the women. As provision of all types of pain relief in labour has staffing implications, there is an urgent need to investigate whether the provision of intrapartum analgesia reflects the needs of the women using the service.

Aberdeen Maternity Hospital has offered a 24-hour epidural service since 1972. In the 1970's and early 80's, high concentration bolus doses of local anaesthetics were used for epidural analgesia, which were administered by anaesthetists rather than midwives. In more recent times infusion regimen, generally consisting of a loading dose of 0.25% Bupivacaine followed by an infusion of 0.125% Bupivacaine with 2μg/ml fentanyl was used. Combined -spinal epidurals are rarely used in the hospital, the few cases on record have been classified as epidurals.

There is a midwives' unit based in the labour ward in the Aberdeen Maternity Hospital, which deals with all uncomplicated deliveries. It however does not offer epidural services, for which a woman needs to be admitted to the main labour ward. This ward is run by both doctors as well as midwives. The hospital has an average yearly delivery rate of 5,000.

The Aberdeen Maternity and Neonatal Databank records and stores information on pregnancies, deliveries and neonatal outcomes of all women residing in the Grampian region of Scotland from the 1950's[5]. Information on the type of analgesia used in labour is available from 1986 at the Databank.

In this retrospective epidemiological study, we aimed to analyse routinely collected data on the use of epidural, opioid and Entonox or no analgesia used for pain relief in labour and delivery in the Grampian region of Scotland in order to analyse their trends over a period of 16 years between 1986 and 2001. We also compared the characteristics of pregnancy, labour and delivery in the women using the different types of analgesia, to investigate their association with different forms of pain relief in labour.

## Methods

This was a population based epidemiological study. Data on all deliveries occurring in the study region over the 16 year period (1986 and 2001) were extracted retrospectively from the Aberdeen Maternity and Neonatal Databank.

There were numerous overlaps between the different types of analgesia used, as women often tried a variety of options during labour. For the purposes of analysis, we have used three groups of analgesia in labour:

1. Epidural – Where the women received epidural analgesia irrespective of any other type of analgesia administered before or during labour and delivery.

2. Opioid – Where the women received morphine, pethidine or diamorphine intramuscularly with or without Entonox (pre-mixed 50%nitrous oxide and oxygen) during labour.

3. No analgesia – Where the women received either no analgesia or Entonox only during labour, irrespective of the type of analgesia used for delivery (i.e. spinal or general anaesthesia for caesarean delivery)

Disaggregated data on those who had Entonox and those who had no analgesia at all were not available from the Databank and have therefore been classified together in the same group.

Data were analysed using Microsoft Excel and Statistical Package for Social Scientists (SPSS) version 13 software.

The rates of the three different types of analgesia in each year from 1986 to 2001 were calculated using SPSS software and graphs of trends were produced using Microsoft Excel.

Maternal characteristics including age, height and weight, pregnancy and labour characteristics, the mode of delivery and neonatal outcomes were compared among the three different analgesia groups.

Continuous data were expressed as mean or median and the differences were assessed by ANOVA, while categorical data were expressed as percentages and were analysed using Chi squared test. A probability value of less than 0.01 was considered statistically significant. The risk-adjusted associations were compared by a binary logistic regression model and were presented as Odds Ratios with 95% Confidence Intervals.

## Results

A total of 81,418 deliveries were analysed. Of these, 12,659 (15.5%) women had epidural, 33,819 (41.54%) had used opioids and 26,974 (33.13%) received either Entonox or no analgesia at all. A total of 7,966 (9.78%) women had elective Caesarean sections under spinal or general anaesthetic before going into labour and were therefore excluded from the subsequent analysis.

### Trends of different forms of intrapartum analgesia

**Figure 1:**
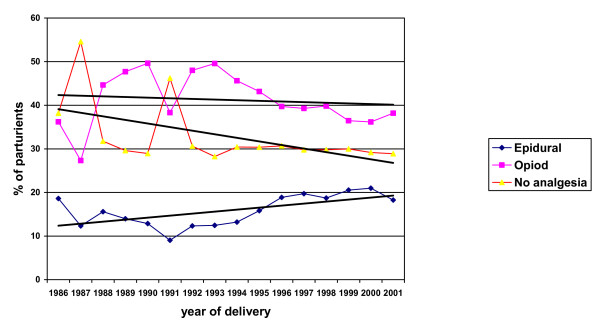
Trends of different types of analgesia used for labour in the Grampian region of Scotland (1986–2001).

Fig. 1 shows the trends in the use of epidural, opioid and no analgesia (or Entonox only) in Aberdeen Maternity Hospital between 1986 and 2001. The uptake of epidural analgesia varied between 9.07% in the year 1991 to 20.99% in 2000, with an average of 15.5% over the whole time period. Opioid analgesia both with and without Entonox was by far the most popular (41.54%). After exponential smoothing to produce trend lines, the use of epidural analgesia showed a slight increase, from 12.35% in 1987 to 18.27% in 2001, while fewer labouring women were using Entonox or no analgesia at all.

### The association between demographic, pregnancy and delivery characteristics and the choice of analgesia in labour using univariate analysis

Table 1 shows the association between epidemiological characteristics and the choice of analgesia in labouring women. The maternal demographic characteristics analysed were the ages of women in complete years at the time of delivery, their height (in cm) recorded at booking for antenatal care, and their weight prior to delivery in Kgs. All three characteristics were found to be significantly different in the three groups. Women receiving epidural analgesia were the shortest (mean height 161.96 cm) and the heaviest (mean weight 64.1 Kg). They also tended to be younger than the women receiving Entonox or no analgesia, but older than those in the opioid group.

**Table 1:** Maternal, pregnancy, labour and delivery characteristics and neonatal outcomes in different types of labour analgesiaData are expressed as mean (standard deviation), median (IQR) or number (percent)

	Epidural	Opioid	No analgesia	Significance*
	n = 12,659	n = 33,819	n = 26,974	
Maternal characteristics*				
Age (years)	27.12(+/- 5.44)	26.99(+/- 5.23)	28.54(+/- 5.17)	**<0.01 (0.0005)**
Height (cm)	161.9(+/- 6.38)	162.5(+/- 6.23)	162.6(+/- 6.21)	**<0.01(0.006)**
Weight (Kg)	64.10(+/-13.37)	63.00(+/-12.58)	62.40(+/-11.93)	**<0.01(0.008)**
**Pregnancy**				
characteristics*				
Primiparas	9457(74.7%)	18882(55.9%)	7190(26.7%)	**<0.01(0.0008)**
Gestational age (weeks)	39.64(+/-1.87)	39.44(+/- 2.24)	38.85(+/-2.32)	**<0.01(0.005)**
Complications	5447(43.03%)	9481(28.03%)	4502(16.69%)	**<0.01(0.0001)**
• Pre-eclampsia	750 (5.9%)	744 (2.2%)	355 (1.3%)	
• APH	1259(9.95%)	3219(9.52%)	2016(7.47%)	
**Labour and delivery**				
characteristics*				
Induced labour	5404(42.69%)	9975(29.49%)	4549(16.86%)	**<0.01(0.0005)**
Duration of labour (hrs)	31.1(+/- 6.3)	20.6(+/- 5.2)	13.6(+/-4.4)	**<0.01(0.0008)**
Instrumental delivery	5461(43.15%)	4879(14.43%)	1746(6.48%)	**<0.01(0.0001)**
Caesarean section	3687(29.13%)	5384(15.92%)	1971(7.31%)	**<0.01(0.0006)**
Neonatal Outcomes*				
Stillbirth rate	41(0.32%)	351(1.03%)	106(0.39%)	**<0.01(0.005)**
Neonatal death rate	27(0.56%)	103(1.35%)	137(0.50%)	**<0.01(0.001)**
Apgar (1 min) 7 or less	59(9.75%)	108(13.52%)	70(5.13%)	**<0.01(0.005)**
Apgar (5 min) 7 or less	148(1.14%)	807(2.37%)	350(1.29%)	**<0.01(0.007)**
Weight of baby (g)	3439.2(+/- 577.9)	3364.4 (+/- 584.1)	3360.5 (+/-607.2)	**<0.01(0.008)**

* Actual P-value presented in parenthesis, all variables were significant at 0.05 significance level

The women receiving the three different types of analgesia also differed in gravidity, length of their gestation period and the incidence of pregnancy related complications like antepartum haemorrhage or pre-eclampsia. Primiparae required more pain relief and therefore commonly chose opioid or epidural analgesia, although even among them parenteral opioids were more popular than epidural analgesia. Women receiving epidural analgesia had the longest period of gestation (mean 39.6 weeks +/- 1.87 Std. Dev). This was 5.5 days longer than the mean gestation period of the women in the no analgesia group, who had the shortest length of gestation. Pregnancy related complications were also more common in the epidural group – recording an incidence of 5447 (43.03%), while it was much less frequent in the opioid group {9481 (28.03%)} and the no analgesia group 4502(16.69%). Albuminuric pre-eclampsia was much more common among the women receiving epidural analgesia {750(5.9%)} than those receiving opioid {744(2.2%)} and no analgesia {355 (1.3%)}. Antepartum haemorrhage was also most common in the epidural group, having an incidence of 1259 (9.95%).

The characteristics of labour in the three groups were all found to be significantly different. Labour was much more commonly induced in the epidural group {5404 (42.7%)} compared with 9975 (29.5%) in the opioid group and 4549 (16.9%) in the no analgesia group. Women choosing epidural analgesia had the longest duration of labour - 31.1 hours versus 20.6 hours in the opioid group and 13.6 hours in the no analgesia group (Table 1). Moreover, the rates of instrumental (43.2%) or caesarean (29.1%) delivery were the highest in the epidural group, in contrast to the rates in the opioid group (14.4% and 15.9% respectively) and in the no analgesia group (6.5% and 7.3% respectively).

With regard to neonatal outcomes, the opioid group had the poorest results. The comparative statistics are presented in Table 1. The stillbirth rate 351(1.03%) was the highest in this group, as was the neonatal death rate 103(1.35%). Moreover, this group had the highest proportions of babies with Apgar Scores of 7 or less, both after 1 minute {108(13.5%)} as well as after 5 minutes {807(2.4%)} of birth. The mean birth weight was the highest in the epidural group (3439.21 gm) and lowest in the no analgesia group (3301.15 gm). These differences were all found to be significant at 1% significance level.

### Results of the multivariate analysis comparing epidural and non-epidural analgesia used in labour

Table 2 shows the results of the binary logistic regression analysis as Odds Ratios with 95% confidence intervals. After adjusting for age, parity, height and weight, the women receiving epidural analgesia were much more likely to have had some pregnancy related complication like antepartum haemorrhage or pre-eclampsia (OR = 2.1, 95% CI 1.82, 2.44). However, the length of gestation or the duration of labour no longer remained significantly different. Labour was 2.8 times more likely to be induced while instrumental delivery was 5 times more common and caesarean section 7 times more so in the epidural group than in the non-epidural group. There was no association detected with stillbirth and the use of intrapartum analgesia. Neonatal death was less likely with epidural than with non-epidural analgesia (OR 0.44, 95% CI 0.15, 0.57). Although babies born of mothers who had epidurals were more likely to have Apgar Scores of 7 or less at 1 minute, this was not true of the same scoring at 5 minutes.

**Table 2:** Risk adjusted association of characteristics between epidural and non-epidural analgesia (Binary logistic regression analysis)

Characteristics	Odds Ratio	95% Confidence Intervals
Gestation period	0.98	0.97 – 1.00
Complications*	2.11	1.82 – 2.43
Induced labour	2.77	2.60 – 2.96
Longer labour	1.01	1.00 – 1.01
Instrumental delivery	5.12	4.99 – 5.15
Caesarean section	7.25	5.66 – 9.29
Stillbirth	0.85	0.51 – 1.42
Neonatal death	0.44	0.15 – 0.58
Apgar (1 min) 7 or less	1.20	1.07 – 1.33
Apgar (5 min) 7 or less	1.00	0.67 – 1.52
Weight of baby	1.00	1.001 – 1.008

*Complications include antepartum haemorrhage and pre-eclampsia

## Discussion

An analysis of the trends in the uptake of a particular service is useful for the future planning of that service. Despite the 24 hour availability of a dedicated labour ward anaesthetist, we found that the uptake of epidural analgesia at the Aberdeen Maternity Hospital to be very low (15.5%). This may have serious implications for future planning of anaesthetic services for the labour ward. Over the 16 years of the study period the rate of use of epidural analgesia has seen very little change, although an upward trend is beginning to occur and is likely to continue in the future. The uptake of epidural services has increased from 12.35% in 1987 to 18.27% in 2001 – an absolute increase of 5.92%. Although it still remains well below the national average of 24% [6] and is less than a third of that in the USA (60%) [7], it signifies an increase of almost 50% in the uptake of epidural services in the region. A survey of obstetric anaesthetic service conducted in Germany in 1996 revealed that the epidural rate was greater than 70% in larger units with more than 1000 deliveries per year [8]. A similar rate was reported from the Tripler Army Medical Centre in Hawaii [9]. The most popular analgesia used in the labour ward of the Aberdeen Maternity Hospital was opioid analgesia with or without Entonox and this has been the intrapartum analgesia of choice throughout the time period of the study, both in primi and multigravidae. This is in contrast to the findings of an American study by Goldberg et al [10], who found epidurals to be the analgesia of choice for nulliparous women.

We found that the characteristics of the women who receive the different types of analgesia were significantly different. The women who received epidural analgesia were younger, shorter and heavier, and delivered larger babies. Almost three quarters of them were primigravidae and were also much more likely to have suffered pregnancy related complications like antepartum haemorrhage and pre-eclampsia. Labour was much more likely to have been induced in these women. Women in this group were 5 times more likely to have an instrumental delivery and 7 times more likely to have a Caesarean section.

Mothers who chose opioid analgesia had the poorest neonatal outcomes in terms of stillbirth rate, neonatal death rate and the Apgar scores at one and five minutes after birth.

Subscribing to the hypothesis that selection of analgesia for labour was not entirely based on women's preferences, we attempted to find the demographic, antepartum and intrapartum factors that determine the use and choice of intrapartum analgesia. A previous similar attempt [11] was made by Dickinson et al to identify the factors influencing the selection of analgesia in spontaneously labouring nulliparous women at term gestation, using prospectively collected data on 497 women. The authors found that women using epidural analgesia were shorter, had longer gestation periods and delivered heavier babies. These findings were all corroborated by the present study, although the practical significance of these differences may be questioned, since they were very small differences in comparison with the standard deviation of the parameters.

All studies including randomized controlled trials have found an increased duration of labour in women using epidural analgesia. We found this to be the case in our dataset on univariate analysis, but after adjusting for confounding factors, this difference was no longer evident. Although most studies have found an increased instrumental delivery rate with epidural analgesia, at least one meta-analysis [12] found that not to be the case. We found a much higher rate of instrumental as well as caesarean deliveries with epidural analgesia in our study. The influence of epidural analgesia on caesarean sections continues to be a topic of debate among researchers and clinicians. Almost all well conducted randomised trials and two recent meta-analyses [1,2] have shown that the caesarean section rate is not increased with the use of epidural analgesia, but observational studies like ours continue to find an association. This is probably due to the fact that women who are at risk of having a Caesarean section are also those who are likely to have more painful labours and therefore tend to use epidural analgesia.

We found the worst neonatal outcomes for opioid analgesia on univariate analysis, but this was not evident when epidural analgesia was compared to non-epidural analgesia in the regression model. This is a surprising finding since one would expect that mothers who chose epidural analgesia are likely to be more sick and therefore have worse perinatal outcomes. We reviewed the literature in search of an explanation. Liu and Sia [13] in their systematic review found no statistical difference in the Apgar Scores of neonates born to mothers using epidural analgesia when compared to those using opioid analgesia. Moreover, Mansoori et al [14] found that the type of intrapartum analgesia had no significant adverse effect on the neonate, at least in the short term, although neonates in the epidural group had worse Apgar Scores at 1 minute compared to those using no analgesia. However, Halpern et al [12] in their meta-analysis concluded that neonatal outcomes were better after epidural than parenteral opioid analgesia, a finding similar to the present study; and the authors speculate that this may be related to the dose and timing of the parenteral opioid injection. As our database extends to the mid nineteen eighties, the neonatal resuscitation techniques may not have been as well developed as they are now; the babies born to mothers who chose opioid analgesia being the quickest to respond to resuscitation.

One other significant finding from our study is the high incidence of pregnancy related complications like pre-eclampsia and antepartum haemorrhage in the women using epidural analgesia. We failed to find a similar association reported in literature, where women with high risk pregnancies were by and large excluded from trials and observational studies.

Most previous studies in the area of intrapartum analgesia have used survey methods to analyze obstetric anaesthetic practice. The main strength of this study is that it is population based and includes a very large dataset (n = 81,418). It is also a longitudinal study over 16 years. To our knowledge, no previous attempt has been made to study analgesia in labour on such a large scale. The Aberdeen Maternity and Neonatal Databank records data on the pregnancies and deliveries of all women residing in a geographical area using stringent coding criteria and therefore provides the ideal setting for conducting longitudinal studies like ours. The data are collected routinely and as the event occurs, therefore minimizing recall bias and virtually eliminating problems of misclassification due to change from one type of analgesia to another.

One of the potential limitations of this study is its inability to analyze disaggregated data on the use of Entonox and no analgesia at all. While very few women were expected not to use any analgesia at all in the later part of the study period, this may not have been so in the earlier years. Therefore, we have had to limit our regression model to a binary logistic one comparing epidural with non-epidural analgesia. This may not give the full picture when considering certain outcomes like stillbirth and other adverse neonatal outcomes, where the univariate analysis shows that the use of opioid analgesia is associated with the worst perinatal outcomes. Other than that, our study suffers from the same limitations of any other case-control study, where adjusting for confounders may be inadequate and observational studies like this will continue to find associations that may not be evident in randomized controlled trials. Moreover, as the analysis of trends in the region shows, the usage of epidural analgesia by women in this hospital is very low, it is obvious that women with high-risk pregnancy (preeclampsia and antepartum haemorrhage) are likely to be recommended epidural analgesia as part of their obstetric management and therefore the dataset is subject to selection bias.

Although the choice of analgesia in labour is supposed to be made by the woman during her pregnancy and childbirth and an epidural service is meant to be available "on demand", several studies have shown that this is not always the case(7, 10). Moreover, although the final decision on the choice of analgesia is made by the woman herself, it is highly influenced by the information provided, which may not always be evidence based. For example, health care providers may believe in the potential association between epidurals and caesarean delivery despite several randomized controlled trials and meta analyses producing evidence to the contrary, and this can influence the information provided to the women. Further work needs to address women's preferences of analgesia for labour pain and the factors influencing these preferences.

## Conclusion

This study shows that in the Grampian region of Scotland between 1986 and 2001, non-epidural analgesia was the most popular choice for pain relief in labour, although an increase in the uptake of epidural services is beginning to occur. The choice of intrapartum analgesia is associated with the epidemiological characteristics of the women's pregnancy, labour and delivery. Further investigations into the reasons for the low uptake and usage of epidural analgesia for intrapartum pain relief in the study region are recommended using both qualitative and quantitative methods.

## Ethics and consent

As this study was carried out using routinely collected anonymized data, formal ethical approval was not considered necessary.

## Competing interests

The author(s) declare that they have no competing interests.

## Authors' contributions

SB was responsible for conceiving the idea for the research project, involved in supervising the study design and statistical analysis and writing the final report. TW was responsible for carrying out the statistical analysis and writing the first draft report; FK gave overall supervision and contributed to the writing of the manuscript and assessing it critically.

## Pre-publication history

The pre-publication history for this paper can be accessed here:


